# How effective is proximal fibular osteotomy in redistributing joint pressures? Insights from an HTO comparative in-silico study

**DOI:** 10.1186/s13018-024-04807-8

**Published:** 2024-06-04

**Authors:** Jorge Eduardo Morales Avalos, Rodolfo Morales-Avalos, Karla V. Martínez-Guajardo, Luis Miguel Pacheco-García, Simone Perelli, Joan Carles Monllau, Antonio J. Sánchez Egea, Gil Serrancoli

**Affiliations:** 1 Department of Mechanical Engineering, Universitat Politècnica de Catalunya, Eduard Maristany 16, 08019 Barcelona, Barcelona Spain; 2 Laboratory of Biomechanics, Articular Physiology and Experimental Orthopedic Surgery, Department of Physiology, School of Medicine, Universidad Autonoma de Nuevo Leon, 64460 Monterrey, Nuevo León Mexico; 3 Department of Orthopedic Surgery and Traumatology, Hospital del Mar, Universitat Autonoma de Barcelona, Pg. Marítim de la Barceloneta, 25, 08003 Barcelona, Barcelona Spain; 4 ICATKnee (ICATME), Hospital Universitari Dexeus, Universitat Autònoma de Barcelona, 08028 Barcelona, Barcelona Spain

**Keywords:** Knee joint, PFO, HTO, In-silico, FEA

## Abstract

**Background:**

Knee osteoarthritis (KOA) represents a widespread degenerative condition among adults that significantly affects quality of life. This study aims to elucidate the biomechanical implications of proximal fibular osteotomy (PFO), a proposed cost-effective and straightforward intervention for KOA, comparing its effects against traditional high tibial osteotomy (HTO) through in-silico analysis.

**Methods:**

Using medical imaging and finite element analysis (FEA), this research quantitatively evaluates the biomechanical outcomes of a simulated PFO procedure in patients with severe medial compartment genu-varum, who have undergone surgical correction with HTO. The study focused on evaluating changes in knee joint contact pressures, stress distribution, and anatomical positioning of the center of pressure (CoP). Three models are generated for each of the five patients investigated in this study, a preoperative original condition model, an in-silico PFO based on the same original condition data, and a reversed-engineered HTO in-silico model.

**Results:**

The novel contribution of this investigation is the quantitative analysis of the impact of PFO on the biomechanics of the knee joint. The results provide mechanical evidence that PFO can effectively redistribute and homogenize joint stresses, while also repositioning the CoP towards the center of the knee, similar to what is observed post HTO. The findings propose PFO as a potentially viable and simpler alternative to conventional surgical methods for managing severe KOA, specifically in patients with medial compartment genu-varum.

**Conclusion:**

This research also marks the first application of FEA that may support one of the underlying biomechanical theories of PFO, providing a foundation for future clinical and in-silico studies.

## Background

Knee osteoarthritis (KOA) is one of the most common degenerative diseases in adult population [1, 2]. One of the primary factors leading to KOA is degeneration of the articular cartilage, which occurs as a result of progressive wear and tear [3–5]. The knee medial compartment is the most commonly affected. Total knee arthroplasty (TKA) [6] is the typical method used to restore knee function in severe cases. However, other surgical interventions have been suggested as an alternative to TKA. The high tibial osteotomy (HTO) [7, 8] is one of the most common surgical interventions for medial compartment early stage KOA. HTO interventions (like open wedge high tibial osteotomy) are reserved for those patients with medial compartment osteoarthritis, associated with genu-varum, young and active individuals, in which delay of a knee prosthesis is desired.

Osteotomies might homogenize the contact pressures. HTO aims to shift the weight-bearing line from the arthritic compartment to the opposite tibiofemoral non-injured compartment [9–11], with a small tendency to increase the load of the lateral compartment [7]. Usually with an average overcorrection of $3^\circ$ valgus [12]. In recent years, some other techniques, such as proximal fibular osteotomy (PFO) [13, 14], have been reported to relieve pain, and facilitates a more neutral alignment of the lower extremity [14–19] in the knee joint (KJ) while improving its functionality [13, 20, 21]. PFO is accompanied by a trend towards redistribution of pressure and stresses on the KJ, from the medial compartment to the lateral and posteriolateral regions of the knee. [22, 23]. In low- and middle-income countries, the acceptance of PFO and its practice is increasing due to the straightforward procedure, the reduced cost, and fast rehabilitation time [24]. However, because PFO is not a standardized surgical procedure, not enough reliable statistical information is available on its effectiveness. Furthermore, the underlying biomechanics of PFO have not been fully described.

PFO is considered a simple, safe, and affordable procedure [25, 26] consisting in removing a section of approximately 2 cm of the fibular bone 6 to 10 cm below the fibular head [14, 15]. The procedure involves cutting through the intermuscular space between the longus extensor digitorum muscle and the longus/shortus peroneus muscle complex [15]. However, it is important to exercise caution during the procedure to minimize the risk of potential complications, specifically the iatrogenic injury to the common peroneal nerve or its branches [19]. In particular, in younger patients, PFO accompanied by comprehensive surgical planning and appropriate indications can be an exceptional procedure for treating medial compartment osteoarthritis [27–29]. Mahadik et al. [17] showed that surgery time was shorter for PFO compared to HTO and observed a pain reduction after the 4-week follow-up in a cohort study of 60 patients. Other studies have shown that certain conditions, such as patients with medial joint space narrowing, are more likely to experience better clinical outcomes after undergoing PFO. This improvement can be seen in terms of alignment and hip-knee-ankle (HKA) angles [8, 24]. However, there is still a lack of long-term data. A recent study [25] showed that severe medial KOA (Kellgren–Lawrence (K–L) grade $= IV$) was significantly associated with patient dissatisfaction after proximal fibular osteotomy over a mid- to long-term follow-up.

There are two main theories that could explain the mechanism of the PFO: (1) the theory of non-uniform settlement [21, 30, 31], which states that the excessive pressures on the medial knee are caused by the increased resistance of the lateral compartment due to the support of the strong trabecular bone of the fibula and (2) the theory of too many cortices [29] which states that the medial condyle is supported by one cortex whereas the lateral condyle is supported by one tibial cortex and two fibular cortices making it difficult to balance loading when the medial side collapses in a varus-deformed knee with an intact fibula. In both, the main determining factors are the biomechanical properties of the proximal fibula with respect to the tibia and the presence of more cortices supporting the lateral side of the knee, respectively.

This study aims to evaluate and compare stress distribution within the affected lower extremity bone structures, and the contact pressure (CP) and center of pressure (CoP) at the KJ contact zones between articular cartilage and the tibial plateau. The analysis was conducted on five patients with severe genu-varum using finite element analyzes (FEA) to simulate an in-silico PFO. To our knowledge this is the first work to analyze the location of the KJ CoP and the distribution of forces preoperatively and postoperatively in patients treated with HTO for medial compartment osteoarthritis and compare it with an in-silico PFO.

## Methods

### Patient demographics

**Fig. 1:**
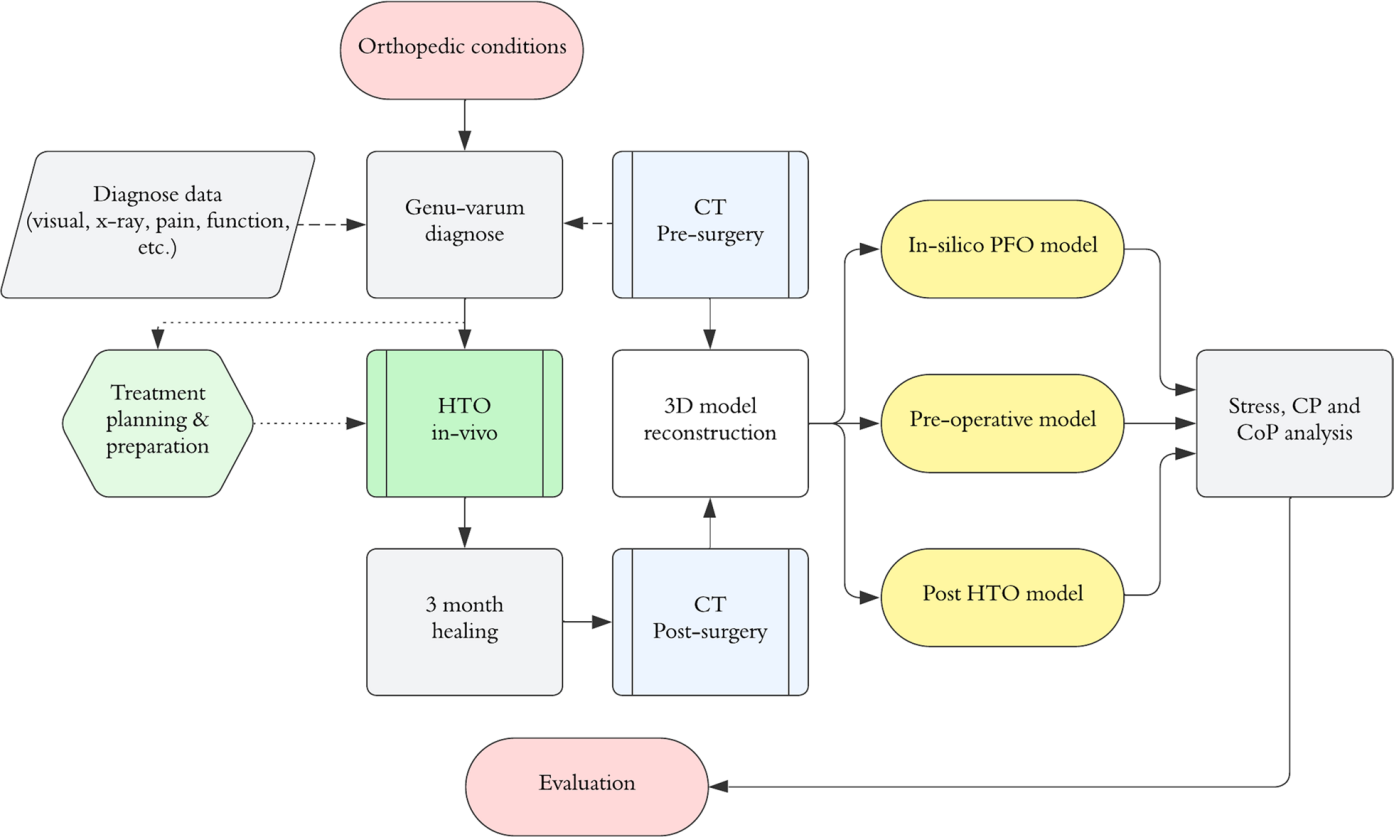
General methodology of the proposed study. Approach to build the pre-surgery, post HTO and post in-silico PFO models

The analysis presented in this study was applied to five patients (four men and one woman, P1–P5) with an average age of $40 \pm 10$ years and a total weight of $80 \pm 10$ kg. All patients self-reported pain on the medial side of the KJ. A lower extremity in each subject was associated with severe varus deformity. All clinical data gathered came from regular medical evaluations and treatments of genu-varum. The patients were informed of the work to accept the use of their retrospective information for scientific purposes. The general methodology followed by this work is illustrated in Fig. 1.

The present protocol was approved by the Institutional Review Board and Research Ethics Committee of the School of Medicine and University Hospital Dr. José Eleuterio Gonzalez of the Universidad Autónoma de Nuevo León (U.A.N.L.) with registration number # FI23-00002.

### Radiographic and clinical examination

The diagnosis and treatment procedures used for this study, according to a knee surgeon co-author of this work, consisted of the following steps:

- Clinical examination
- Imaging observation by means of X-ray (anteroposterior, lateral, axial and Rosenberg views) and computer tomography (CT) scans of the complete lower limb, from the head of the femur down to the distal tibia. The CT was taken with the patient in the supine decubitus position with knee flexion at 20 degrees.
- Arthroscopy performed before surgical intervention to confirm the degeneration stage of the cartilage and verify the menisci for ruptures.
- Application of the HTO, with a three-months recovery and healing phase
- Clinical examination and confirmation using X-ray and CT scan after the three-months healing process.

The medial-proximal-tibial angle (MPTA) was measured using full-length standing anteroposterior radiographs. This angle was found to be altered in all cases. The lateral-distal-femoral angle was found to be within the normal ranges in all of the patients. Hence, the deformities were considered to be of tibial origin. For each diagnosis, it was verified that: (a) there was no osteoarthritis present in the lateral and femoropatellar compartments of the knee; (b) the patellar height was normal; and (c) there were no menisci ruptures or chondral-associated injuries (in that case these would be repaired at the time of the HTO surgery). In terms of postoperative care, proprioceptive loading of 5–10% with crutches was allowed and free movement from the first day with the use of an extension brace at night. Isometric quadriceps exercises were allowed from day 1. The load was progressively increased from the third postoperative week to allow full weight bearing at 6 weeks postoperatively.

To have a baseline of the original state of each subject, the mechanical axes of the affected limb (mLDFA and mMPTA), the HKA and the total mechanical misalignment were measured according to [4, 32–39] and are presented in Table 1. The Fujisawa method was employed to determine the requisite degree of correction [12]. The patients were classified using the Ahlbaeck classification, with three patients classified as grade 2 and two patients classified as grade 3.

**Table 1:** Preoperative state

Patient	mLDFA (°)	mMPTA (°)	HKA (°)	Varus deformity (°)
P1	91.8	80.7	168.8	11.1
P2	92.6	76.6	164.0	15.9
P3	90.4	78.9	168.5	11.5
P4	90.7	82.9	172.2	7.7
P5	91.5	81.2	169.7	10.2

Relevant tibiofemoral angles, and varus deformity. mLDFA stands for mechanical lateral-distal-femoral angle and mMPTA for mechanical medial-proximal-tibial angle

Six weeks following surgery, postoperative telemetry was conducted. In four of the five patients, a neutral corrected angle of zero was achieved, while in one patient, it was brought to a two-degree valgus. Following the HTO procedure, the patients no longer exhibited signs of pain.

The core of this study relies on the investigation of the effects of PFO compared to HTO by assessing the following variables:

- CP distribution in the lateral tibial cartilage (LTC) and medial tibial cartilage (MTC), before surgery, post HTO and in-silico PFO.
- Anatomical location of CoP relative to the anatomical knee center in all three analyzed conditions.

The location of the center of the knee was measured according to the methodology described in [32]. Consequently, the center of the knee is relative to each patient case and relative to its surgical state (preoperative, post HTO, and post-PFO).

### Original postoperative condition model

Three 3D-models of the lower limb were generated for each subject to analyze the biomechanical behavior of the patients undergoing HTO and to compare the effects of an in-silico PFO. A preoperative and an in-silico PFO model were created using preoperative geometric data, while an HTO model was created using post-surgery data. The detailed model construction process can be found in Appendix Fig. 6.

The preoperative geometry consists of 3D anatomical data extracted from a full-leg CT scan of each patient. The DICOM image data extracted was processed using the Mimics Research 21® software package (Materialise, Leuven, Belgium). After segmentation, geometry post-processing was performed using 3-Matic ® (Version 21, Materialise, Leuven, Belgium). Specifically, 3D models consist of hard structures such as the cortical and trabecular femur, tibia, and fibula bone. Soft tissues include the LTC, MTC, lateral and medial meniscus and the lateral, medial, anterior and posterior crucial ligaments (LCL, MCL, ACL and PCL, respectively). The patella was not considered due to the lack of significant load influence in the normal human standing position over the KJ [40]. The cortical bone of each component was modeled with a constant wall thickness of 2 mm, based on a constant density approach and using the median values proposed by [41–44]. Using a constant thickness for cortical structures as a controlled variable helped to establish the relationships between the variables explored [45]. The trabecular bone was modeled using 3-Matic by filling the cortical cavity with a uniform volumetric mesh.

The preoperative 3D models were carefully assembled and positioned using Catia 3D experience (Dassault Systèmes, Velizy-Villacoublay, France). This process was conducted using frontal plane radiographs previously taken during the diagnostic phase as a visual aid. Continuous contact was ensured between the femur, femoral cartilage, meniscus, tibial cartilage and tibia [46–48], in this order. Ligaments were modeled to ensure proper contact conditions with the other structures, based on the literature [49–55]. In addition, the proximal and distal anterior and posterior tibiofibular ligaments were constructed as 1D linear elements [56, 57].

### In-silico PFO and HTO models

The PFO model was created from the pre-surgery model by removing a 2 cm segment of the fibula bone, 6 cm from its head [14, 15, 17, 24, 29, 45].

The HTO models for each patient were developed using a similar approach to the pre-operative model mentioned earlier. Additionally, a proximal tibial locking plate (TomoFix Osteotomy System, DePuy Synthesis, West Chester, PA, USA) and five fixing screws were required to simulate the HTO implants. The 3D models of the Tomofix plate and screws were obtained from the hospital’s instrumental supplier, where the interventions took place. Implants were assembled and positioned according to their anatomical location and orientation, using postoperative frontal and sagittal X-rays in conjunction with the segmented models.

### Finite element analyzes

To simulate the models response to the weight bearing load distributed over the KJ on a standing position, a 3D implicit single step nonlinear-static FEA was performed using Ansys Mechanical 2022 R1 (Ansys Inc., Pennsylvania, USA) software. Fifteen FEA were developed for this study. Three for each subject: preoperatively, post HTO, and in-silico PFO. Since this is the first study to investigate the PFO influence over the CoP, we believe that a static analysis must be established first in a position where the CP reaches around its maximum [58].

#### Material properties

Material properties were assigned accordingly to our previous studies [45] and based on the literature (all material properties used can be found in Appendix Table 3). All bones were assumed to be 3D rigid bodies, homogeneous, isotropic, and linearly elastic [50, 54]. In addition, ligaments were modeled as 1D linear elements [56, 57] to represent their anisotropy [49, 59].

#### Boundary conditions

Distal ends of both the tibia and fibula were fixed in all studied models [22, 56, 60], mimicking contact interactions with the ankle joint.

A total force of 750 N was exerted vertically on the proximal head of the femur to replicate the complete weight of an average adult weighing 75 kg [39, 48, 54, 55, 61–66].

At the same location, a remote displacement condition was applied. This allowed the femoral bone to change position and orientation on the vertical and anteroposterior axes, simulating the movement of the hip [67].

#### Contact interactions

Surface-to-surface contact interfaces were created at the component system level to ensure proper tissue interactions [68]. Trabecular bone structures were bonded to cortical bone at the entire contact interface. In addition, the inner surface of the femoral cartilage was bonded to the distal surface of the femur. An augmented Lagrangian contact algorithm [54] with a pinball region radius of 0.1 mm [69] and frictional behavior with a friction coefficient of 0.2 [22] was used to model the interface between the femoral cartilage, the meniscus, and the upper surface of the tibial cartilage. Furthermore, the distal surface of the tibial cartilage was bonded to the cortical bone of the tibial plateau. Ligaments were bonded to their structures in contact. Contact between the fibula and tibia was defined as bonded using a 3 mm pinball radius to compensate for the gap generated between the bones during segmentation. To model the interfaces between the tibia plate and the fixing screws, interactions were implemented with a friction coefficient of 0.3 for the tibia-plate and 0.2 for the tibia-screws contact pairs [70]. The locking screws of the HTO plate were simulated to rigidly bond with the holes in the plate.

#### Mesh generation

All bone and soft tissue structures were discretized into tri-linear tetrahedral elements consisting of 10 nodes. This method is particularly suitable for complex components, as it accurately represents proximity and curvature, highlighting critical areas. This is especially beneficial when working with soft materials, as discussed in [71]. Hexahedral elements were used to discretize the added implants.

Sensitivity analyzes were conducted to assess the potential impact of mesh density on the results. The maximum CP within the tibial cartilages was used as a measure to evaluate any significant changes in the stress distribution. Table 2 provides a detailed description of the different mesh sizes used for each structure type. Case D (as shown in Table 2) was determined to be the most optimal case, whose peak stress change was lower than 5% and based on its balanced convergence time

## Verification of the FEM model

To verify our simulation reference model, we found that the stress distribution at the whole tibial bone and the tibial plateau before and after PFO was similar, as reported by [22] and following the results of a recently published study [45]. Furthermore, as far as we are aware, only [56] have reported CP values following PFO, and a similar trend in CP distribution can be observed between the osteotomised model and the PFO osteotomised model of this work. However, no CoP coordinates have been previously documented after a PFO in-silico intervention. Nevertheless, the CP results at the knee joint presented by [68] are consistent with our CP distribution and values for the reference model.

## Results

### Whole limb stress distribution

**Fig. 2:**
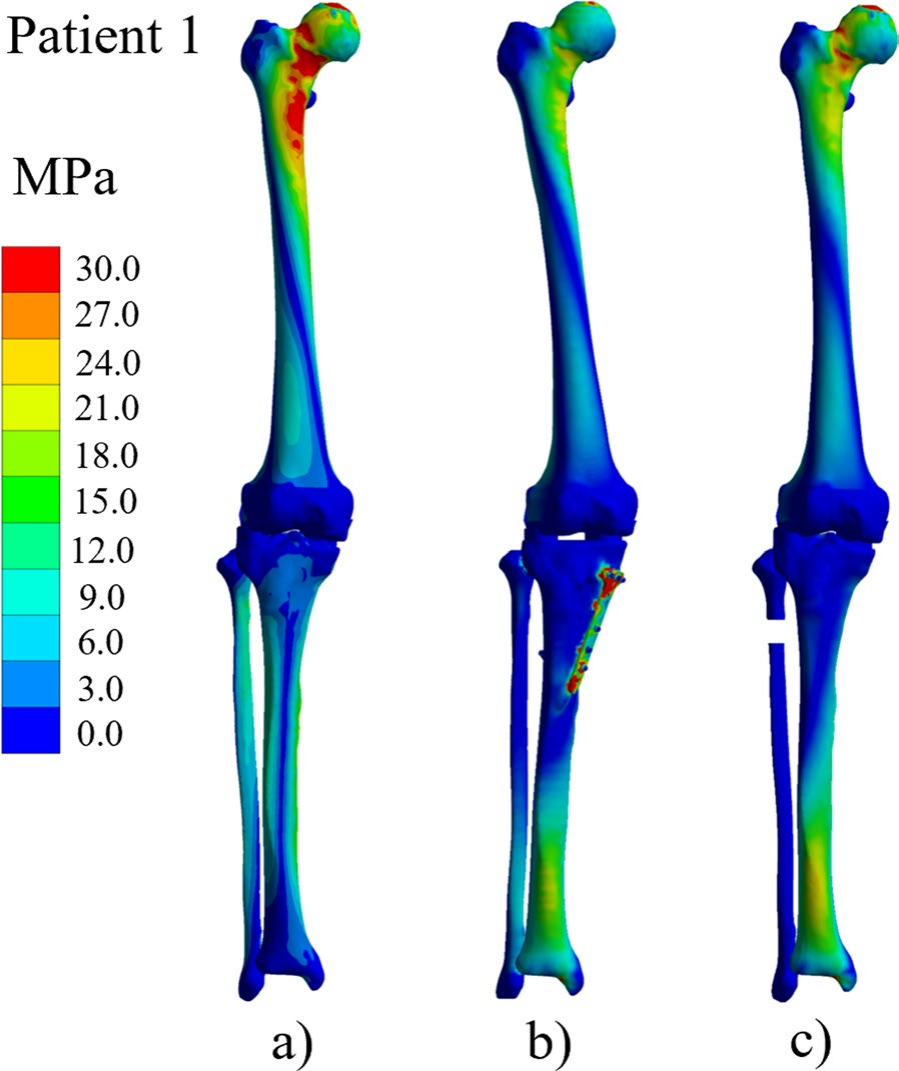
Von Mises Stress distribution on the affected lower extremity under normal natural standing position. From left to right: **a** pre-surgery, **b** post HTO and **c** in-silico PFO

A more uniform distribution of stress concentration was observed following both HTO and PFO surgeries. An example of the von Mises stress distribution for the entire lower limb is shown in Fig. 2 (Patient 1, P1). Prior to HTO, a high stress concentration can be seen in the head and neck of the proximal femur (see Fig. 2a). The maximum principal stress (MPS) (found at the cortical bone of the femoral head and neck) decreased from 29.3 MPa pre-surgery (Fig. 2a) to 21.5 MPa after the HTO (Fig. 2b) and to 27.0 MPa after in-silico PFO (Fig. 2c). In accordance with previously reported results [45], the femoral shaft and neck after PFO tend to be released from stress after the in-silico PFO.

Prior to HTO, the medial compartment and the entire shaft of the tibia report higher stress concentrations in the medial zone (see Fig. 2a) than post HTO and in-silico PFO. The MPS at the tibial bone was 22.5 MPa (medial mid-shaft, Fig. 2a), 19.7 MPa (anterior mid-shaft, Fig. 2b) and 24.1 MPa (anterior mid-shaft Fig. 2c), pre-surgery, post HTO and post in-silico PFO, respectively. At the fibular bone, 4.7 (proximal and distal), 10.4 (distal-anterior) and 1.3 MPa (distal-anterior). The absence of the support given by the fibular bone shifts the loads to the tibial bone. The tibial MPS after PFO corresponds to about a 10% increase from the original pre-surgery state. Post HTO, this value decreased to around 10%.This trend was observed across all patients.

### Force transmission at the tibia-fibula

**Fig. 3:**
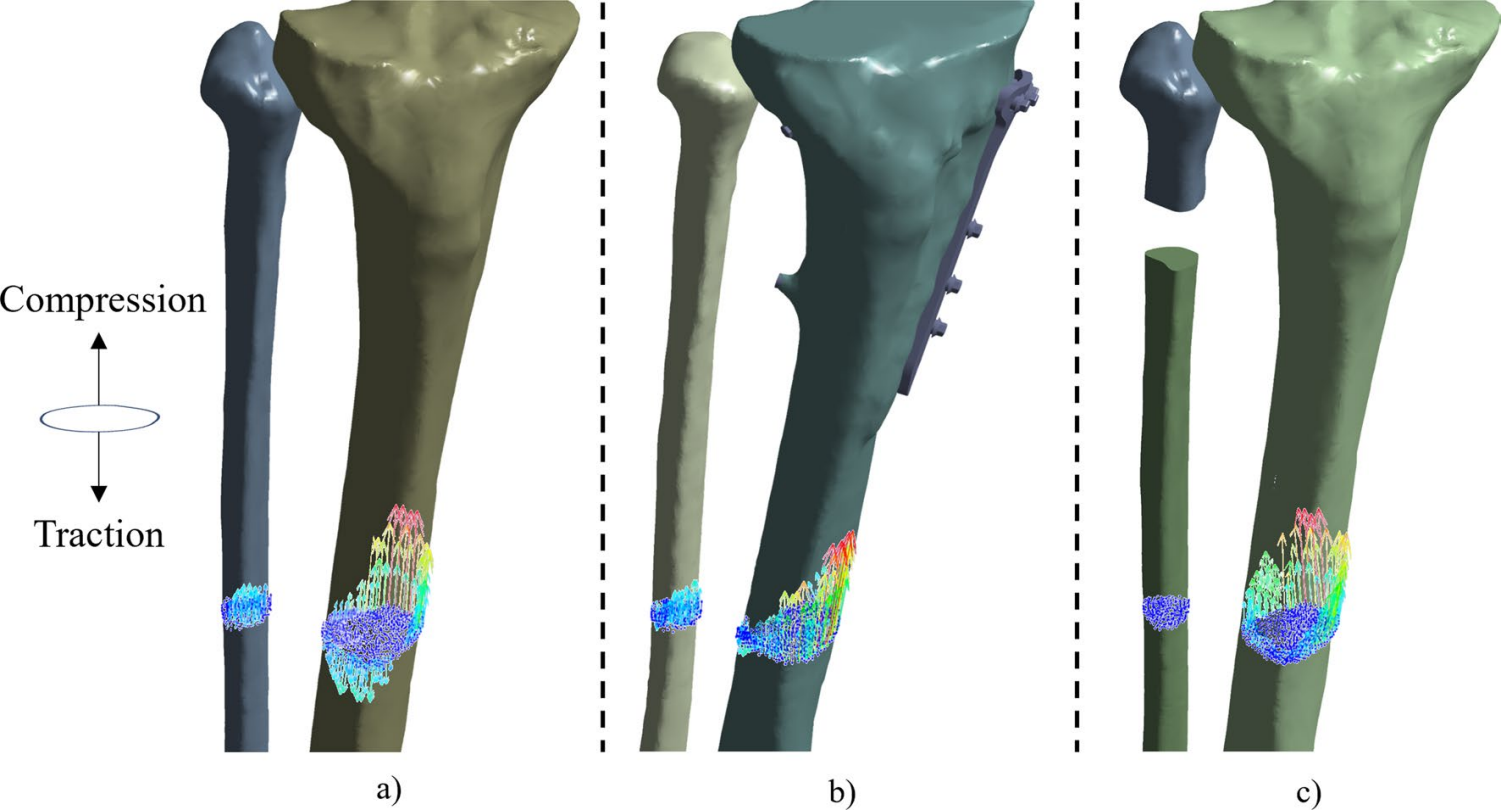
Patient 3 reaction forces at the middle of the tibial and fibular shaft applied to the proximal section; **a** pre-surgery, **b** HTO and **c** in-silico PFO. The length and color of the force arrow symbols are relative to the resultant reaction force value. The sum of the force vectors is equal to the applied load (750 N)

Reaction analyzes at a section 160 mm from the fibula-tibia junction revealed differences in the transmission of forces at both the fibula and tibia among all three cases (see Fig. 3). Pre-surgery, the lateral part of the tibia was under traction, while the fibula and the medial part of the tibia were under compression. Post HTO, the forces transmitted to the tibia were homogenized, being the whole section under compression. After PFO, a similar effect is observed at the tibia, being the medial tibial forces higher than in HTO showing a tendency to redirect forces towards the posteriolateral region of the knee joint.

### Contact pressure distribution and CoP

**Fig. 4:**
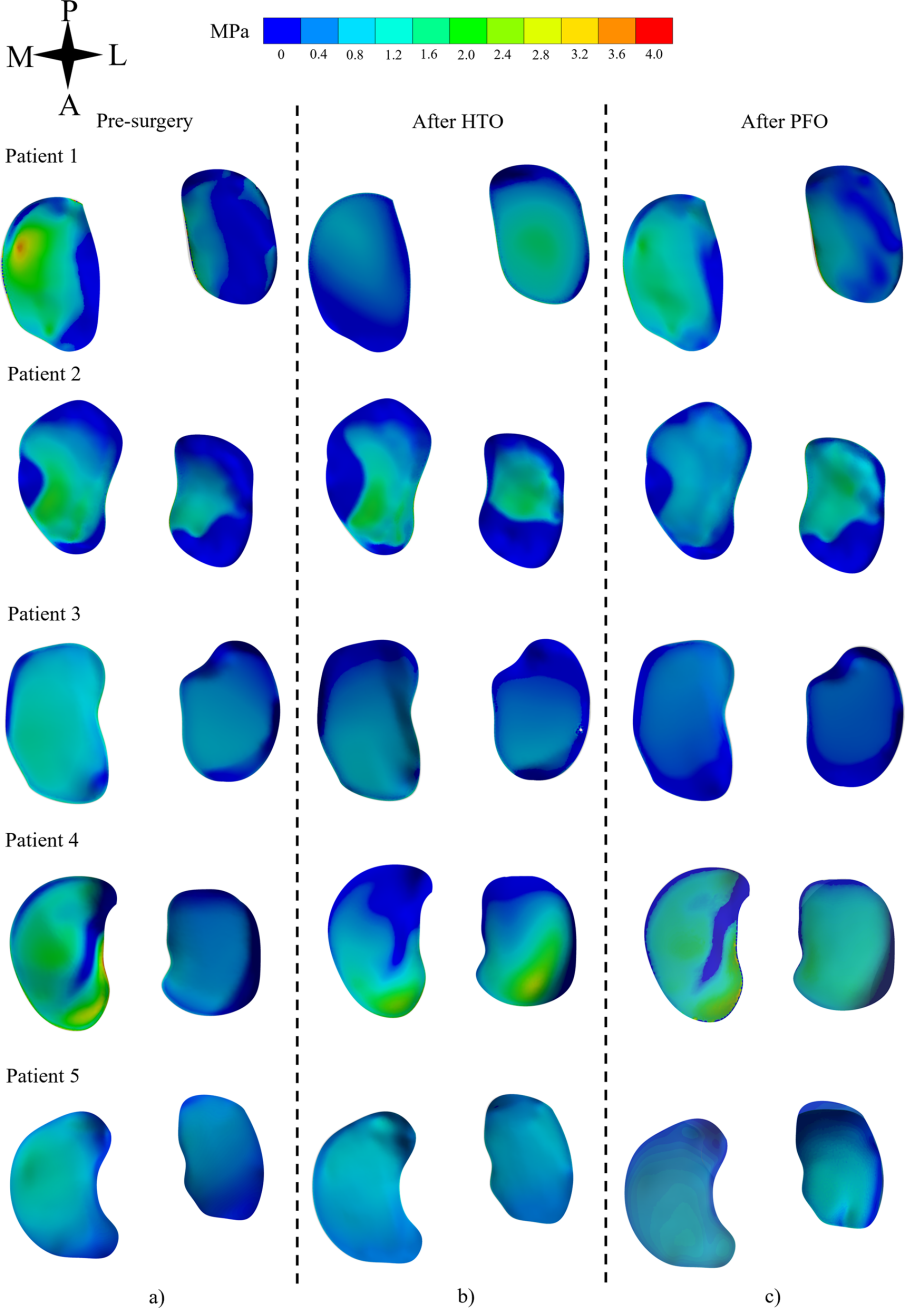
Pressure distribution at the tibial plateau-cartilage contact interface, **a** before the surgery, **b** post HTO and **c** after PFO. *M* medial, *L* lateral, *P* Posterior, *A* Anterior

Before the surgery, and as commonly associated with genu-varum diagnosed patients, higher CP can be observed towards the medial compartment of each patient (see Fig. 4a), with average values ranging from 2.3 to 2.5 MPa.

Post HTO, the CPs were homogeneously distributed along both compartments of the knee (see Fig. 4b and Table 4). A similar trend is observed after the in-silico PFO (see Fig. 4c). Medial compartment CP was reduced across all patients post HTO (1.148 MPa ($SD = \pm 0.39$)) as well after in-silico PFO (1.128 MPa ($SD = \pm 0.09$)). However, pressure values at the lateral compartment were increased post HTO (1.146 MPa ($SD = \pm 0.073$)) and after in-silico PFO (1.126 MPa ($SD = \pm 0.066$)). The average CP distribution ratio for both post surgery cases (HTO and PFO) was found to be almost 1:1 between MTC and LTC (see Table 4).

The CoP was calculated using the total pressure values computed for the FEM mesh nodes at the tibial plateau contact interaction zone with each of the tibial cartilages as defined in Eq. .

1 $$\begin{aligned} CoP_x = \frac{\Sigma X \cdot P_t}{\Sigma P_t} ,\quad CoP_y = \frac{\Sigma Y \cdot P_t}{\Sigma P_t} ,\quad CoP_z = \frac{\Sigma Z \cdot P_t}{\Sigma P_t} \end{aligned}$$

**Fig. 5:**
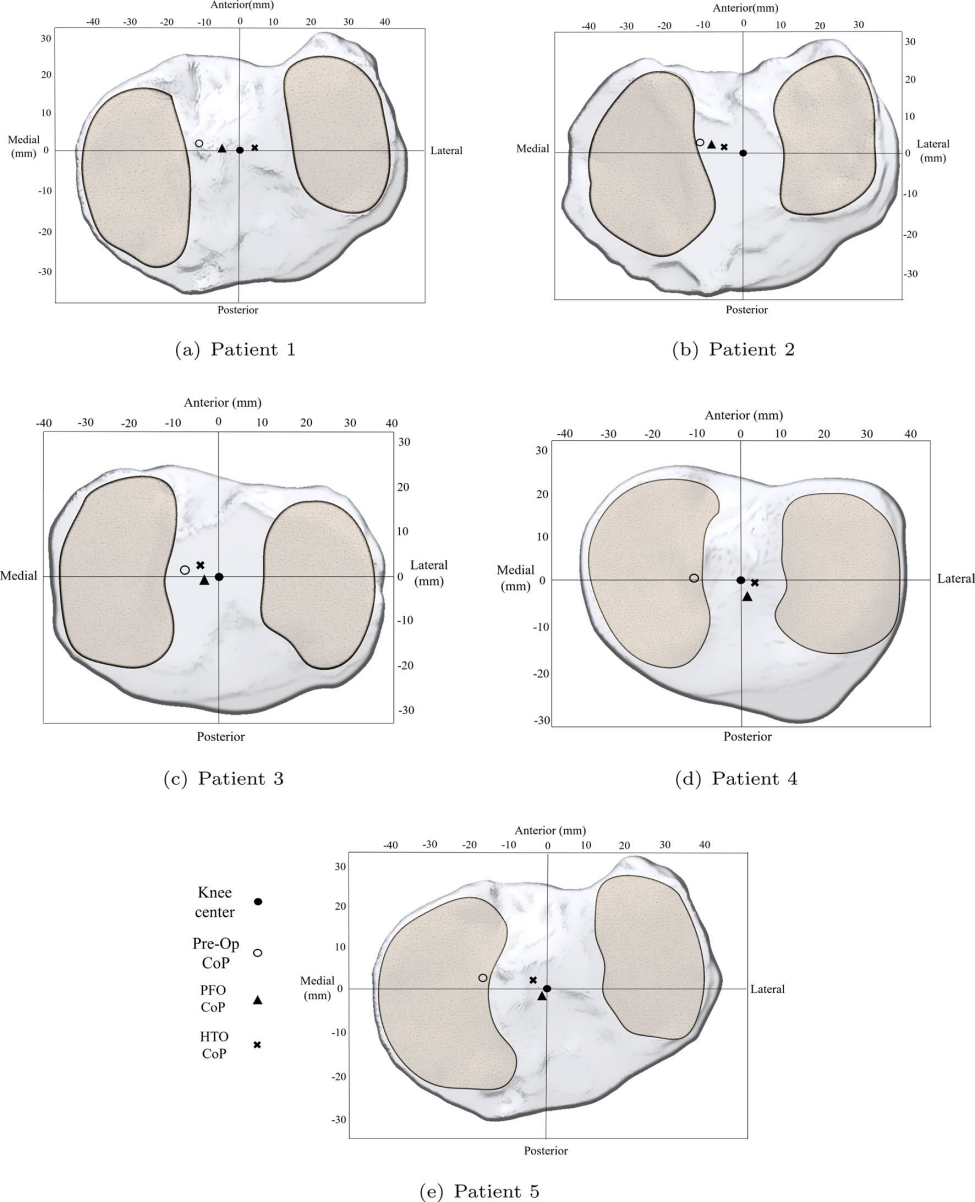
CoP anatomical location for the studied cases at the tibial plateau

After both HTO and PFO surgeries, the CoP’s deviation from the center of the knee was reduced. In all patients, the preoperative CoP was concentrated in the medial compartment of the knee as seen in Fig. 5, with values ranging from 7.7 to 16.7 mm in the medial to lateral direction and from 0.5 to 2.7 mm in the anterior to posterior direction. These values represent the original anatomical deviation of the CoP before surgery (see Table 4). Following HTO, the CoP tends to return to or approximate its position relative to the center of the knee. Similarly, after in-silico PFO, a comparable effect can be observed, with the CoP moving closer to the center of the knee.

## Discussion

The principal findings of this study reveal that following PFO there is a consistent trend of relocating the CoP within the medial and lateral compartments towards the center of the knee. For all patients, the pressure in the medial compartment induced by varus deformation was alleviated after performing PFO. These results align with findings obtained from analyzes of the effects of HTO in other studies [17, 54].

Our work analyzed the effects of PFO on the CP distribution of the KJ, including its CoP and whole-limb stress distribution. This study is the first to compare these parameters in a preoperative original state model, a postoperative HTO model, and a simulated PFO model. Compared to the few studies [22, 23, 56, 57] that have utilized FEA to describe the effects of PFO on stress changes for KOA with varus deformity, this is the first to investigate the influence on CoP anatomical location after an in-silico PFO. Furthermore, such effects have never been compared to clinically intervened patients using HTO as a treatment. Significant changes in the CP at the tibial plateau and stress distribution of the whole-limb were found after PFO. Analysis of the healed models of the HTO-intervened patients revealed that this behavior was very similar and, in some cases, the deviation of CoP after PFO was lower than the one calculated post HTO.

Pressure reduction in the medial compartment may result in an immediate reduction in pain, as reported in some clinical studies [14, 28, 72, 73] and supporting recent studies findings such as [16, 25, 74]. Based on the few published studies, PFO promises to be safe (i.e. bleeding amount was found to be reduced in comparison to HTO [17]), inexpensive, and implant-free, as a treatment against medial compartment KOA ([21]).

The results observed in our analysis are in line with the non-uniform settlement [21, 30, 31]. Releasing the support of the fibula on the varus knee, the transmitted forces accommodate on the tibia and get more homogenized through the whole section of this element (Fig. 3). Before the surgery, major compression forces were transmitted by the medial side of the tibia, consistent with the varus malalignment and the principal compressive stresses [38] being concentrated in the medial compartment of the knee. Note that some literature studies mention that the density of the fibula might be higher than the tibia in KOA [28, 75], so the observed effect would even be higher. After both PFO and HTO, a compression force is observed in the lateral part of the tibia (see Fig. 3b, c). The ’too-many-cortices’ theory could be reconsidered. Following PFO, the number of cortices on the lateral side is reduced from three (two fibular and one tibial support) to one (solely tibial), as seen from a frontal view. The results indicate that having three cortices during varus malalignment patients the lateral part of the tibia to traction. However, following PFO application, this side of the tibia experiences compression.

The absence of clinical evidence reporting CP values or CoP coordinates of the knee joint after PFO, direct validation of our comparative study cases of varus/valgus deformation cannot be made. A clinical study by [76] has reported that PFO is only suitable for patients with mild varus deformity ($\le 5^\circ$) and less effective for those with severe varus deformity ($\ge 5^\circ$), which aligns with the results and findings presented in this work. Fu et al. [25], found that among the 203 patients who had undergone PFO, those with an HKA angle $(^\circ )$ of $173.5 \pm 4.5$ were satisfied with the post-operative results while those with an HKA angle $(^\circ )$ of $170.2 \pm 5.7$ were unsatisfied after undergoing PFO. The results of our study indicate that the degree of varus deformation affects the effectiveness of PFO. Specifically, it impairs the ability of the CoP to shift to a neutral position following surgery.

One key limitation of this FEA study is the restricted boundary conditions, limiting bone movement range. Consequently, it is not possible to quantify the extent of misalignment correction after in-silico PFO. However, we can still observe and measure the trend of CoP displacement after load redistribution at the KJ. Figure 5 illustrates how this parameter can serve as a useful indicator of PFO effectiveness, depending on the subject’s initial conditions.

The similarity in the CoP location after in-silico PFO and post HTO results indicates that the local relative deviation (measured in x and y coordinates) calculated to the center of the knee results in a similar tendency between PFO and HTO to redistributes pressures in the knee joint neutrally. Consequently, the outcomes of this study do not depend on the amount of correction achieved through HTO, but on the level of deviation calculated from each surgical state CoP and the center of the knee. As illustrated in Fig. 5. The objective of this study was not to assess the efficacy of in-silico PFO measured as the malalignment correction compared to HTO. Rather, the aim was to compare the tendencies of the change in pressure distribution and the CoP re-localization after in-silico PFO when compared to post HTO. The efficacy of PFO was assessed by analyzing the joint pressure distribution, not the malalignment correction. It is assumed that relying on the degree of malalignment correction as a basis for the results will lead to an increased bias in the distribution of contact pressures within the knee joint.

Another limitation is its reliance solely on static simulations, which narrows understanding of PFO’s efficacy in real-life scenarios. Future studies should integrate dynamic simulations to mimic basic human actions, like walking, offering in-silico biomechanical insights for gait analysis as reported by [23]. However, the highest knee CP is achieved when the foot touches the ground during walking [77]. Therefore, the CP distribution at the KJ in our study should remain similar.

This study provides a valuable understanding of the biomechanics and effectiveness of PFO as a treatment for medial compartment genu-varum. The confidence of the medical community in PFO as a viable treatment option will only increase with the availability of robust clinical and scientific evidence. This in-silico study, which uses HTO results as a baseline, represents an important step towards building a stronger evidence base for using PFO in treating medial compartment genu-varum.

## Conclusion

The stress and CP distribution effects of the PFO models proposed in this work were compared to the HTO analysis, demonstrating similar agreement. The resection of the fibular bone in the presence of genu-varum deformities leads to a redistribution of the CoP towards the center of the knee, a phenomenon comparable to what is observed post HTO. Analyses of the reaction forces at the level of the bone component before surgery, post HTO and after in-silico PFO provide further insights into the underlying mechanisms of the PFO described by the nonuniform settlement and the too many cortices theories.

## Figures and Tables

**Fig. 6:**
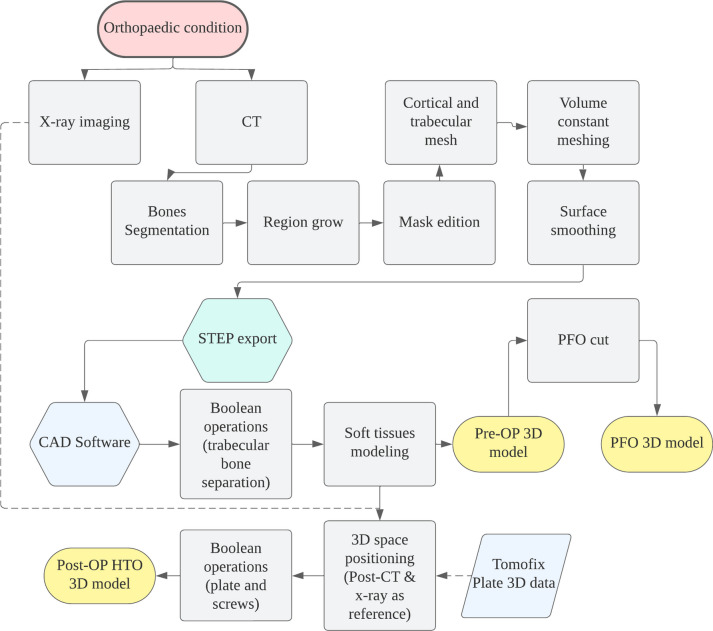
Pre surgery (original), in-silico PFO, and post HTO model construction methodology

## Data Availability

The datasets used and/or analyzed during the current study are available from the corresponding author on reasonable request.

## Appendix 1: Additional tables

Conducted FEM mesh element size sensibility analyses. Case D was selected as optimal between mesh density, stress change and convergence time (Table 2).

**Table 2:** Sensitivity analysis for the different KJ components (ES = element size (mm), NOE = number of elements)

Cases	Cortical bone	Trabecular bone	Soft-tissues	Stress change (%)
	ES–NOE	ES–NOE	ES–NOE	–
A	10–16,097	10–10,635	5–3327	12.16
B	5–39,268	7.5–18,688	3–9050	6.83
C	3 –95,185	5–50,352	1 -140,466	3.28
D	1–2,062,113	3–211,294	0.5–1,056,473	2.87
E	0.5–15,341,556	1–5,436,767	0.25–8,173,879	1.63

Cortical bone properties following [78]. Trabecular bone after [79]. Meniscus and cartilage as a combination of [54, 80]. For the ligaments, different sources were investigated, such as [50, 59, 81, 82]. The implants were modeled following the properties given by [11] (Table 3).

**Table 3:** Material properties of bone, cartilage, ligaments, meniscus and implants

Material	E-module (MPa)	Poisson’s ratio	Density ($kg/m^3$)	Stiffness (N)	$\varepsilon _L$
Cortical bone	16,800	0.3	2000	–	–
Trabecular bone	840	0.2	1350	–	–
Meniscus	59	0.49	1000	–	–
Cartilage	5	0.46	1100	–	–
ACL	345	0.4	1000	5000	0.03
PCL	345	0.4	1000	9000	0.03
LCL	345	0.4	1000	2000	0.08
MCL	332.2	0.4	1000	2750	0.03
Ti	110,000	0.3	4500	–	–

The mean values for contact pressure across all patients for the medial and lateral compartments, along with the location of the CoP, are presented in Table 4.

**Table 4:** Mean CP (± standard deviation) values and CoP coordinates presented at the tibial cartilages for all patients and cases

Patient	Case	LTC CP (MPa)	MTC CP (MPa)	CoP (x,y) (mm)
P1	Original	$0.25\pm 0.27$	$2.46 \pm 0.36$	(11.2,2.6)
	PFO	$1.31\pm 0.23$	$1.31\pm 0.20$	(4.5,0.1)
	HTO	$1.46 \pm 0.23$	$1.24 \pm 0.12$	(4.1,0.9)
P2	Original	$0.19\pm 0.23$	$2.40\pm 0.29$	(11.9,2.7)
	PFO	$1.36\pm 0.28$	$1.28\pm 0.14$	(8.3,2.2)
	HTO	$1.26\pm 0.27$	$1.34\pm 0.29$	(5.0,1.9)
P3	Original	$0.28\pm 0.12$	$2.50\pm 0.15$	(7.9,1.6)
	PFO	$1.37\pm 0.14$	$1.45\pm 0.15$	(3.5,− 0.9)
	HTO	$1.32\pm 0.17$	$1.46\pm 0.21$	(4.9,2.6)
P4	Original	$0.24\pm 0.19$	$2.56\pm 0.12$	(10.3,0.5)
	PFO	$1.35\pm 0.26$	$1.27\pm 0.23$	(1.5,− 4.0)
	HTO	$1.45\pm 0.35$	$1.23\pm 0.17$	(3.0,− 0.6)
P5	Original	$0.17\pm 0.13$	$2.42\pm 0.23$	(16.7,2.7)
	PFO	$1.37\pm 0.19$	$1.29\pm 0.14$	(1.3,− 1.8)
	HTO	$1.39\pm 0.12$	$1.33\pm 0.14$	(3.7,1.9)

## Appendix 2: Additional figure

See Fig. 6.

## Abbreviations

KOA: Knee osteoarthritis
TKA: Total knee arthroplasty
HTO: High tibial osteotomy
PFO: Proximal fibular osteotomy
KJ: Knee joint
HKA: Hip-Knee-Ankle
CP: Contact pressure
CoP: Center of Pressure
FEA: Finite Element Analysis
CT: Computer tomography
MPTA: Medial-proximal-tibial angle
LDFA: Lateral-distal-femoral angle
mMPTA: Mechanical medial-proximal-tibial angle
mLDFA: Mechanical lateral-distal-femoral angle
LTC: Lateral tibial cartilage
MTC: Medial tibial cartilage
